# Association between Oral Pathology, Carotid Stenosis, and Oral Bacterial DNA in Cerebral Thrombi of Patients with Stroke

**DOI:** 10.1155/2021/5402764

**Published:** 2021-09-07

**Authors:** Olli Patrakka, Helena Mehtonen, Sari Tuomisto, Juha-Pekka Pienimäki, Jyrki Ollikainen, Heini Huhtala, Tanja Pessi, Niku Oksala, Terho Lehtimäki, Jorma Järnstedt, Mika Martiskainen, Pekka J. Karhunen

**Affiliations:** ^1^Department of Forensic Medicine, Faculty of Medicine and Health Technology, Tampere University and Fimlab Laboratories, Tampere, Finland; ^2^Medical Imaging Center, Department of Radiology, Tampere University Hospital, Tampere, Finland; ^3^Vascular Centre, Tampere University Hospital, Tampere, Finland; ^4^Department of Neurology, Tampere University Hospital, Tampere, Finland; ^5^Faculty of Social Sciences, Tampere University, Tampere, Finland; ^6^Hemorrhagic Brain Pathology Research Group, Tampere University, Tampere, Finland; ^7^Department of Clinical Chemistry, Faculty of Medicine and Health Technology, Tampere University, Fimlab Laboratories and Finnish Cardiovascular Research Center Tampere, Tampere, Finland; ^8^National Institute for Health and Welfare, Helsinki, Finland

## Abstract

**Methods:**

Thrombus aspirates and control arterial blood were taken from 71 patients (70.4% male; mean age, 67.4 years) with acute ischemic stroke. Tooth pathology was registered using CT scans. Carotid stenosis was estimated with CTA and ultrasonography. The presence of bacterial DNA from aspirated thrombi was determined using quantitative PCR. We also analyzed the presence of these bacterial DNAs in carotid endarterectomies from patients with peripheral arterial disease.

**Results:**

Bacterial DNA was found in 59 (83.1%) of the thrombus aspirates (median, 8.6-fold). Oral streptococcal DNA was found in 56 (78.9%) of the thrombus aspirates (median, 5.1-fold). DNA from *A. actinomycetemcomitans* and *P. gingivalis* was not found. Most patients suffered from poor oral health and had in median 19.0 teeth left. Paradoxically, patients with better oral health had more oral streptococcal DNA in their thrombus than the group with the worst pathology (*p* = 0.028). There was a trend (OR 7.122; *p* = 0.083) in the association of ≥50% carotid artery stenosis with more severe dental pathology. Oral streptococcal DNA was detected in 2/6 of carotid endarterectomies.

**Conclusions:**

Stroke patients had poor oral health which tended to associate with their carotid artery stenosis. Although oral streptococcal DNA was found in thrombus aspirates and carotid endarterectomy samples, the amount of oral streptococcal DNA in thrombus aspirates was the lowest among those with the most severe oral pathology. These results suggest that the association between poor oral health and acute ischemic stroke is linked to carotid artery atherosclerosis.

## 1. Introduction

Cardiovascular and cerebrovascular diseases are one of the leading causes of death and disability worldwide, with acute ischemic stroke alone resulting in over 5 million deaths annually [1, 2]. Carotid artery atherosclerosis has been found to be an important cause of large vessel disease and ischemic stroke [3, 4]. Traditional stroke risk factors are hypertension, hypercholesterolemia, diabetes mellitus, smoking, and obesity [1]. Periodontal disease has been found to be a new risk factor for stroke, with the direct mechanism still remaining unclear [5, 6]. In Finland, dental infections are common with the prevalence of caries being almost 100%,and severe periodontitis 15-20% and periapical lesions up to 27% [7]. It has been suggested that oral infections contribute to carotid artery intimamedia thickness leading to carotid artery stenosis and subclinical atherosclerosis [8]. A significant association between tooth loss levels and carotid artery plaque prevalence has been reported [9].

In our recent research [10], we found that DNA typical for bacteria of oral origin can be found in cerebral thrombi of acute ischemic stroke patients. DNA of bacteria typical for both endodontic and periodontal infections have earlier been found in cerebral aneurysms [11], in thrombus aspirates of patients with acute myocardial infarction [12] and in lower limb arterial and deep venous thrombosis [13]. In all these studies, the most frequently found bacterial DNA belonged to the *Streptococcus mitis* group, which belongs to viridans group streptococci. Viridans streptococci species are a known cause of infective endocarditis [14]. However, we do not know what the origin of streptococci in thrombus aspirates is. Bacteria may gain access to the systemic circulation via the root canal of infected teeth or through periodontal pocket and may end up in a thrombus because viridans streptococcal species possess thrombogenic properties [15, 16]. As odontogenic infection has been associated with the development of carotid artery disease and rupture of the atherosclerotic plaque, it may be possible that streptococci originate from complicated carotid plaques [17].

In this study, we applied dental radiography as well as carotid radiological imaging to examine whether oral health and carotid artery stenosis are related to the amount of oral bacterial DNA found in cerebral thrombi using radiologic data of acute ischemic stroke patients. We also analyzed the presence of oral bacterial DNA in an unrelated series of carotid endarterectomies from symptomatic patients.

## 2. Materials and Methods

### 2.1. Subjects

The series comprises 71 acute ischemic stroke patients who were treated by intra-arterial thrombectomy between November 2013 and January 2017 in Acute Stroke Unit of Tampere University Hospital (Table 1). A neurologist (J.O.) examined all patients when they arrived to the hospital and evaluated the possibility of revascularization using thrombectomy together with a neurointerventional radiologist (J.-P.P.) based on clinical symptoms and computed tomography angiography (CTA). All patients have been treated in Acute Stroke Unit of Tampere University Hospital according to modern medical standards, and all methods were carried out in accordance with relevant guidelines and regulations. The degree of carotid stenosis was estimated using CTA or ultrasonography which was performed to 58 (81.7%) of the patients. The etiology of the brain large vessel occlusion of patients treated with endovascular thrombectomy during the study period in Tampere University Hospital was cardioembolic in 38% and atherosclerotic in 62% of the patients (personal communication, Dr. Jyrki Ollikainen). There were no patients with coagulation disorders or undetermined stroke etiology. Thrombus was aspirated from M1-segment of the middle cerebral artery in most of the patients (*n* = 70; 98.6%). The only excluding criteria for recruiting patients were unsuccessful retrieval of the thrombus using mechanical thrombectomy. The median delay time between onset of an ischemic stroke and hospital arrival was 2 h 30 min (range, 0-16 h). Medical history was collected from the Tampere University Hospital digital patient archives. Criteria for dyslipidemia were fP − kol − LDL > 3.0 mmol/L, fP − kol − HDL < 1.0 mmol/L, or fP − Trigly > 2.0 mmol/L.

Carotid arteries from 6 patients of the Tampere Vascular Study (TVS) were obtained (N.O.) aseptically during open carotid endarterectomy in 2005–2009 from symptomatic patients with hemodynamically significant (≥50%) carotid stenosis. Control samples from two patients were obtained from the left internal thoracic artery (LITA) during coronary artery bypass due to symptomatic coronary artery disease. All open vascular surgical procedures were performed at the Division of Vascular Surgery and the Heart Center at Tampere University Hospital.

### 2.2. Imaging and Assessment of Dental Pathology Using Brain Computed Tomography

Noncontrast computed tomography scans (Lightspeed VCT, GE Medical Systems, United States) were taken of all patients when arrived to the hospital to exclude intracranial hemorrhage, which were used for the assessment of oral health. The parameters of those CT scans were as follows: slice thickness of 0.63 mm, field of view 320.0 mm, voltage of 120 kV, and current of 649 mA. With Philips Brilliant™ Workspace (Philips Healthcare, The Netherlands), CT images were reconstructed into 1 mm multislice axial, sagittal, and coronal planes. Additionally, synthetic panorama and 1 mm multislice reconstructions of the right and left oblique sagittal and slice series in accordance with dental arches were reconstructed.

CT reconstructions were inter and intraobserved by two evaluators, an experienced (J.J.) and a trainee oral and maxillofacial radiologist (H.M.) using Carestream Vue PACS software (Carestream Health, United States) and diagnostic monitors (Barco, Belgium). The assessments were performed in dim lighting. Scores that differed between the observers were jointly assessed. In total number of teeth, number of missing teeth, and per each tooth, the following parameters were registered: periapical condition, horizontal and vertical alveolar bone loss, furcation lesions, and condition of pericoronal spaces and caries.

The condition of a tooth was first registered as sound, filled, caries with pulp exposed or teeth being decayed as residual root, but due to the resolution and artefacts deteriorating diagnostic accuracy, we ended up combining the groups as sound/filled and caries with pulp exposed/residual roots. Periapical infection was registered if an osteolytic lesion was found surrounding the root apex. Vertical/angular defect ≥ 1/3 of the root length was considered as vertical bone loss. Scores for horizontal bone loss were difficult to assess from CT scans and because scores differed between evaluators, they were left out from the analyses. Furcation lesion was registered if an osteolytic finding at the furcation was found. Pericoronal space was defined to be infected if the pericoronal space was ≥3 mm, and/or surrounding bone showed signs of infection.

Combined pathology sum was calculated to assess the overall oral health status in the same way as previously used [18–20]. The number of vertical bone defects as well as periapical, furcation, caries, and pericoronitis lesions was summed per each tooth. The patients were then divided into three equal groups based on the sum per each tooth: “normal to slight pathology” (*n* = 21, sum 0-0.06), “moderate pathology” (*n* = 21, sum 0.07-0.23), and “severe pathology” (*n* = 22, sum 0.24-2.00). Edentulous patients (*n* = 5) were excluded from the analysis.

### 2.3. Mechanical Thrombectomy and Thrombus Sample Collection

Mechanical thrombectomy and thrombus sample collection were performed by a neurointerventional radiologist as previously described [10]. An introducer sheet was placed into the femoral artery. A blood sample for background analysis was collected through the sheet. Guiding catheter up to 9 Fr (Merci® Concentric medical) with a tip balloon was navigated into the carotid artery proximal to the occluded site. The microcatheter (0.021” Trevo) with the guide wire was used to navigate through the occluded site and to deploy the stent retriever (Trevo®, Stryken neurovascular) over the thrombus. An additional distal access catheter was used to achieve the thrombus if needed. Forceful aspiration through proximal catheter was acquired with a 60 cc syringe while retrieving the deployed stent. Different device settings were selected by the operator selectively case by case. Thrombectomy was repeated until the angiological result of satisfaction. Gathered thrombus was divided into 1.5 cc Eppendorf for quantitative PCR analysis and a histological sample part in a formalin container.

### 2.4. Quantitative PCR

The presence of bacterial DNA was identified using qPCR with the ABI PRISM 7900 HT Sequence Detection System (Applied Biosystems, Foster City, Calif) [21] with Maxima Probe/ROX qPCR MasterMix (Thermo Fischer Scientific, Waltham, Mass). Arterial thrombus aspirates were compared with arterial control blood samples, as opposed to venous to venous to reduce any potential bias caused by sampling from different sites and, subsequently, bias resulting from different conditions like flow dynamics and pressure. The presence of bacterial DNA in the thrombus and in control blood samples was determined by using published primers and a probe for *Streptococcus* spp., mainly *S. mitis* group, *A. actinomycetemcomitans*, and *P. gingivalis* using human housekeeping gene, RNAseP (Applied Biosystems), as a reference gene [12]. The positive controls were reference bacteria from ATCC collection (*Streptococcus mitis* ATCC 49456, *A. actinomycetemcomitans* ATCC 700685, *P. gingivalis* ATCC 33277, LCG Standards AB, Borås, Sweden). Each measurement was performed as duplicates or quadruples in uncertain cases. DNA was extracted from the entire thrombus in most of the cases. If the aspired thrombus was large, a small part of it was taken and sent to histological analysesm and DNA was extracted from rest of the thrombus. The relative amounts of bacterial DNA in the samples were calculated by the comparative threshold cycle (Ct) method (*ΔΔ*Ct, *Δ*Ct_sample_ − *Δ*Ct_control_) [12, 22–25], where the sample was a thrombus aspirate. and the control was a blood sample from the same patient. First, the differences of the Ct values (*Δ*Ct) between candidate bacteria and reference gene measurement (Ct from candidate bacteria − Ct from RNAseP) for each sample were calculated; then, the comparative Ct (*ΔΔ*Ct) (*Δ*Ct from thrombus−*Δ*Ct from patients own arterial blood) was calculated. The samples were separated into two different groups: samples were marked negative if 2^−ΔΔCt^ < 2 and positive if 2^−ΔΔCt^ ≥ 2 [26, 27]. The presence of bacterial DNA in carotid atherosclerotic plaques and healthy control LITA samples was studied using the same comparative method by keeping a LITA Ct values as a reference.

### 2.5. Statistical Analysis

Associations between bacterial DNA findings and nominal dental parameters were analyzed using Pearson's chi-square test. Age-adjusted logistic regression analysis was used to estimate the odds ratio (OR) and 95% confidence interval (CI) for associations between bacterial DNA findings, grade of carotid artery stenosis, and combined dental pathology. The number of teeth was not normally distributed, and therefore, median values and quartiles (Q1 and Q3) were calculated. Statistical significance was set at *p* ≤ 0.05, and analyses were done using IBM SPSS Statistics for Windows, Version 27.0. Armonk, NY: IBM Corp.

## 3. Results

### 3.1. Patient Characteristics and Bacterial DNA Findings

There were 50 (70.4%) men and 21 (29.6%) women in the study population. The mean age of the patients was 67.4 years. None of the patients had been treated with antibiotics or experienced severe dental infections or septicemia during the stroke. Using universal bacterial primers, bacterial DNA was found in 59 (83.1%) of the thrombus aspirates with a median of 8.6-fold rate compared to the control peripheral blood sample from the same patient. Viridans streptococcal DNA was found in 56 (78.9%) of the thrombus aspirates (median, 5.1-fold). All thrombi were negative for both *P. gingivalis* and *A. actinomycetemcomitans* bacterial DNA. One arterial blood sample was positive for *P. gingivalis,* and two arterial blood samples were positive for *A. actinomycetemcomitans* bacterial DNA. All characteristics of patients are presented in Table 1. Carotid stenosis was found from 54 (81.2%) patients and carotid dissection from 4 (5.6%) patients. There were 11 (19.0%) patients with ≥50% stenosis and 43 (74.1%) patients with <50% stenosis.

### 3.2. Association between Dental Pathology and Bacterial DNA Findings in Thrombus Aspirates

**Figure 1:**
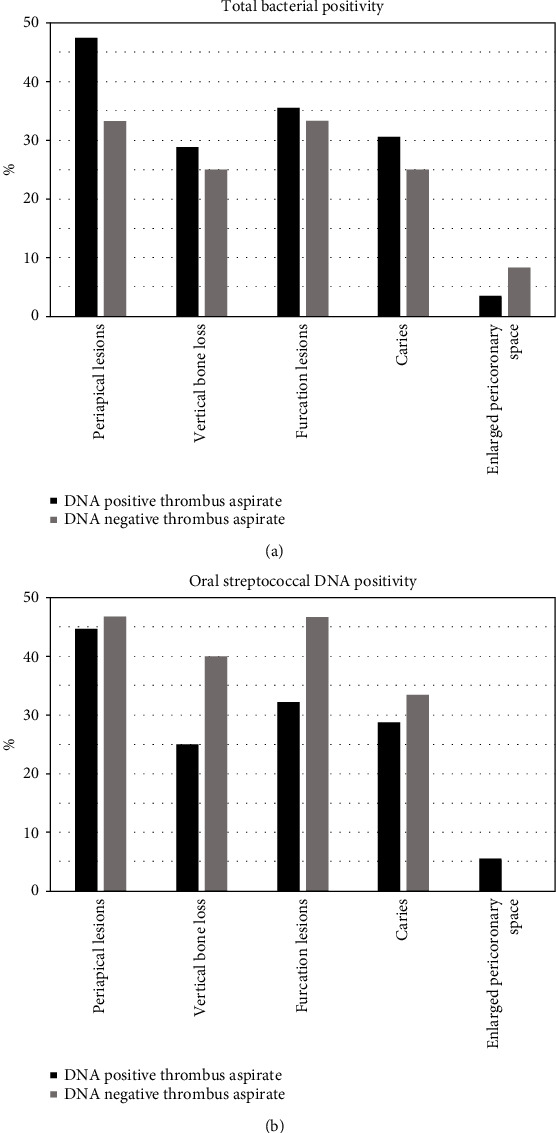
Dental pathological lesions in patients with or without total bacterial DNA (a) and oral streptococcal DNA (b) findings in thrombus aspirates. All pairwise comparisons *p* > 0.05.

**Figure 2:**
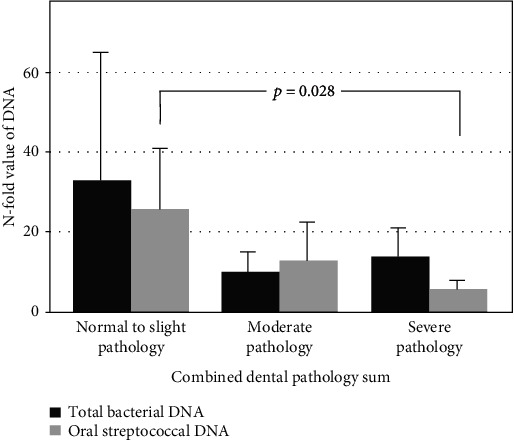
*N*-fold value of total bacterial (*p* = 0.197) and oral streptococcal (*p* = 0.032) DNA between the thrombus and arterial blood from the same patient and the relationship with combined dental pathology sum. (a) Normal to slight pathology (*n* = 21). (b) Moderate pathology (*n* = 21). (c) Severe pathology (*n* = 22).

Patients had poor oral health. They had in median 19.0 (Q1: 9.0; Q3: 26.0) teeth (range, 0-32 teeth). Of the total 71 patients, periapical lesions were found in 32 (45.1%), vertical bone loss in 20 (28.2%), furcation lesions in 25 (35.2%), caries in 21 (29.6%), and enlarged pericoronal spaces in 3 (4.2%) patients. There was no association between the number of teeth and bacterial DNA counts in thrombus aspirates. Connection between dental pathology and bacterial DNA findings in thrombus aspirates was not statistically significant when dental variables were treated individually (Figure 1). However, when pathologies were summed, a linear correlation between oral streptococcal bacterial DNA findings and tooth condition was found (*p* = 0.032) in age-adjusted analysis, whereas this association was not found for total bacterial DNA amounts (*p* = 0.197). In age-adjusted analysis, patients with better oral health had more oral streptococcal bacterial DNA in their thrombus than the group with the worst pathology (*p* = 0.028). On mean, the group with normal to slight pathology had 25.8-fold difference, the moderate pathology group had 13.0-fold difference, and the severe pathology group had 5.8-fold difference between thrombus and arterial blood oral streptococcal DNA amount (Figure 2).

### 3.3. Association between Carotid Artery Stenosis and Bacterial DNA Findings in Thrombus Aspirates

**Figure 3:**
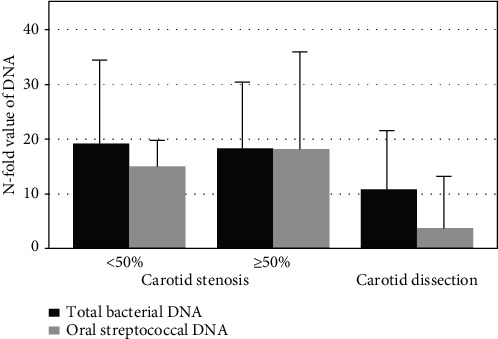
*N*-fold value of total bacterial DNA (*p* = 0.983) and oral streptococcal DNA (*p* = 0.701) between the thrombus and arterial blood from the same patient and the relationship with <50% (*n* = 43) and ≥50% (*n* = 11) carotid artery stenosis. DNA amounts were lower in patients with carotid dissection (*n* = 4).

There was no association between carotid stenosis and total bacterial DNA amount in thrombus aspirates. However, patients with ≥50% stenosis had slightly more (18.1- v.s. 13.9-fold) oral streptococcal DNA in thrombus aspirates compared to patients with <50% stenosis, but this difference was not statistically significant (*p* = 0.578) in age-adjusted logistic regression analysis, due to high variation in the DNA folds and small number of the cases (Figure 3).

### 3.4. Association between Carotid Stenosis and Dental Pathology

**Figure 4:**
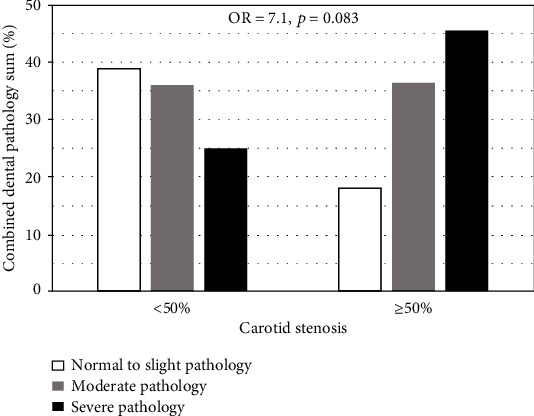
Association between severity of carotid artery stenosis and combined dental pathology. In logistic regression analysis with age and amount of oral streptococcal DNA as covariates, there was a trend (OR 7.1; 95% CI 0.78-65.5; *p* = 0.083) in the association of ≥50% carotid stenosis with more severe dental pathology.

There was no difference (*p* = 0.604) in the number of teeth between those with ≥50% carotid stenosis (18.7 ± 8.9) compared to those with less than <50% stenosis (17.0 ± 10.1). We found that there were more cases (45.5% v.s. 25.0%) with severe dental pathology among patients with ≥50% carotid stenosis compared to those with less than <50% stenosis, respectively. In logistic regression analysis with age and amount of oral streptococcal DNA as covariates, there was a trend (OR 7.122; 95% CI 0.78-65.5; *p* = 0.083) in the association of ≥50% carotid stenosis with more severe dental pathology, while age (OR 1.044, *p* = 0.295) or amount of streptococcal DNA (OR 1.023, *p* = 0.188) was not significant covariates (Figure 4).

### 3.5. Oral Bacterial DNA in Carotid Stenosis Samples

Oral streptococcal DNA (viridans group streptococci, mainly *Streptococcus mitis*) was detected in 2 (33%) of the 6 surgically collected sterile atherosclerotic carotid endarterectomy samples showing advanced atherosclerosis. In one of these cases (17%), DNA from *P. gingivalis* was also amplified. Cases negative for oral bacterial DNA also showed severe atherosclerosis.

## 4. Discussion

It has been reported that carotid artery atherosclerosis is an important cause of large vessel stroke, and a part of acute ischemic stroke may be due to embolism from the carotid arteries [3, 4, 28]. It has also been found that periodontal disease and fewer teeth may be associated with an increased risk of ischemic stroke [5]. We found recently that most thrombus aspirates from acute ischemic stroke patients contained DNA from oral streptococcal bacteria [10]. In the present study, we found that stroke patients had poor oral health. We found an inverse association of the severity of oral pathology but a positive trend of carotid artery stenosis with the amount of streptococcal DNA in thrombus aspirates of stroke patients. Patients showing more severe carotid stenosis tended to have the worst oral health. We found streptococcal DNA in 1/3 of carotid endarterectomy samples from surgical patients suffering peripheral artery disease.

The most frequent bacteria in cerebral thrombus aspirates in our study, viridans group streptococci, are common oral bacteria associated with the development of dental plaque [29]. Oral streptococci may initiate or contribute to platelet aggregation in coronaries [15]. We have earlier reported viridans group streptococcal DNA in thrombus aspirates of patients with acute myocardial infarction [12] and in lower limb arterial and in deep venous thrombosis [13]. Lockhart et al. demonstrated in 2008 that dental operations such as tooth extraction and daily toothbrushing can cause transient bacteremia. Most translocated bacteria into the circulation that could be cultivated were viridans streptococci [30].

In our study, 60.6% of the patients suffered from moderate or severe dental pathology and had 19.0 teeth left on median. The number of teeth is similar to the numbers in a Finnish national survey, where the average amount of teeth was 17.0 in people aged 65-74 [31], while in same aged Swedish population, the average number of remaining teeth is 26.0 [32]. Although the number of dentists does not differ significantly between Sweden and Finland, the practice of preferring tooth extraction instead of treatment of caries applied in Finland may explain the differences in the number of teeth [7]. Interestingly, stroke prevalence in Finland (395.9 strokes per 100,000 inhabitants) is higher compared to Sweden (368.6 strokes per 100,000 inhabitants) [33], even though the countries share a similar economic and social model as well as a similar health care system [34].

Our findings on the association between bad oral health and low bacterial DNA findings in thrombus aspirates of the same patients might be explained by studies reporting that the longer the periodontal disease exposure, the fewer focal infection focuses there are present. With only few teeth left in the oral cavity as a result of a long-term periodontal disease, the infectious pathway is closing and the continuous flow of oral bacteria through the ulcerated epithelium of gingival periodontal pockets reaching the bloodstream decreases [9, 35]. There are several previous studies that have shown the association between ischemic stroke and tooth loss, especially in younger age groups [5, 6, 36–38]. In our study, where the mean age of the patients was 67 years, we found no association between tooth loss and bacterial DNA counts in thrombus aspirates. Among elderly people, tooth loss is not found to be connected with atherosclerotic vascular diseases [6, 39, 40]. Our patients' tooth number did not significantly differ from the same aged Finnish population. In addition, edentulousness is related to lowered oral bacteria (*P. gingivalis*) IgG levels [41].

We found streptococcal DNA in 1/3 of carotid endarterectomy samples from surgical patients suffering peripheral artery disease. Previously, the presence of odontogenic bacteria, such as viridans group streptococci *(S. sanguinis*) and *P. gingivalis*, has been shown in the atherosclerotic plaque of human carotid artery histologically and by polymerase chain reaction [42, 43]. It has been proposed that bacteria present in carotid artery plaques contribute to the enhanced risk of plaque rupture leading to thrombosis [17]. Viridans group streptococci have been found to be the most common gram positive bacteria persisting intracanal disinfection procedures and after root canal treatment [44]. Koren et al. showed that an abundance of *Veillonella* sp. and *Streptococcus* sp. in the oral cavity was linked to their abundance in carotid atherosclerotic plaques [45].

We found that the association of ≥50% carotid stenosis to be related to more severe dental pathology. Our findings are in line with previous studies [9, 46, 47]. Periodontitis has been shown to elevate the overall infectious burden in generally healthy populations [48], apical periodontitis is suggested to be associated with increased levels of systemic inflammation [49], and chronic low-grade oral infection and inflammation have been related to unfavorable systemic cardiovascular effects [50–52]. Thus, bad oral health may contribute to the progression of atherosclerotic lesions via circulating chemical mediators [53–55].

Our patients with ≥50% stenosis had slightly more oral streptococcal DNA in their aspirated thrombi compared to those with <50% stenosis. We may hypothesize that oral bacterial DNA found in thrombi are originated from ruptured carotid artery plaques. There is evidence of oral bacterial inflammation to be related to the development of the atherosclerotic plaque by the inflammatory mechanism in the arterial wall and through cytokine activation [56–59].

There may be other explanations for our findings concerning oral health and bacterial DNA findings in aspirated thrombus. Extraction of teeth can be done for other reasons than disease related. Temporal sequence is hard to determine. American Heart Association stated in their systematic review that observational studies do not support a causative relationship between periodontal disease and atherosclerotic disease [60]. The relationship has been proposed to be due to different reasons, such as tooth extraction for other reasons than periodontal disease, a change in diet after tooth loss, selection bias, and biological and behavioral factors [61, 62]. Nevertheless, there is a positive linear association between oral health and overall mortality [63–65]. One of the limitations of our study was that we had a small sample size. The grade of carotid artery stenosis was measured using either computerized tomographic angiography or Doppler ultrasound, of which concordance is 79% [66]. We only had two categories of carotid stenosis. Tooth conditions were estimated from CT reconstructions. We did not have the possibility of conducting a clinical examination. Dental and periodontal disease can be recognized with dental CT [67]; yet, it is not as accurate as the golden standard cone-beam computed tomography [68]. In addition, the PCR method we used detects the presence of bacterial DNA in the examined samples but is unable to separate living bacteria from phagocytized bacterial DNA.

## 5. Conclusions

Stroke patients had poor oral health. We found an inverse association of the severity of oral pathology but a positive trend of carotid artery stenosis with the amount of streptococcal DNA in thrombus aspirates. Our results propose that the association between poor oral health and acute ischemic stroke is linked to carotid atherosclerosis. The question in which way oral bacteria are involved in the pathogenesis of acute ischemic stroke or are they solely bystanders remain still open and should be evaluated in forthcoming studies.

## Figures and Tables

**Table 1:** Patients' characteristics.

All patients *N* = 71	
Age (mean ± SD)	67.4 ± 12.5
Male gender, *n* (%)	50 (70.4)
Diabetes, *n* (%)	12 (16.9)
Dyslipidemia, *n* (%)	29 (45.3)
Arterial hypertension, *n* (%)	38 (53.5)
Coronary heart disease, *n* (%)	14 (20.0)
Cerebrovascular disease^a^, *n* (%)	18 (40.9)
Pulmonary disease, *n* (%)	4 (5.71)
Renal insufficiency, *n* (%)	6 (8.57)
Atrial fibrillation, *n* (%)	47 (66.2)
Heart failure, *n* (%)	9 (12.7)
Arrival time to the hospital, hours (median)	2.20

^a^Data for previous cerebrovascular disease was available only for 44 patients.

## Data Availability

The datasets generated and analyzed during the current study are not publicly available due to the individual person's data that are involved but are available from the corresponding author on reasonable request.
